# HIS AND HER EVERYDAY LIFE: GENDER DIFFERENCES IN OLDER ADULTS’ SOCIAL AND PHYSICAL ACTIVITY

**DOI:** 10.1093/geroni/igz038.225

**Published:** 2019-11-08

**Authors:** Meng Huo, Karen L Fingerman, Yee To Ng

**Affiliations:** The University of Texas at Austin, Austin, Texas, United States

## Abstract

The literature links social integration to better physical health, but little research asks how contact with diverse social partners influences older adults’ physical activity in a daily context. We examined this link using the Daily Experiences and Well-being Study and explored whether this link varied by gender. The sample included 175 older women and 138 older men who reported their contact with close partners (e.g., family/friend) and not-close partners (e.g., acquaintances) throughout each day across 5 days. Participants also wore Actical accelerometers to track physical activity. Multilevel models revealed significant gender differences. Older men had reduced physical activity when having contact with close partners, whereas older women maintained physical activity during such contact. Both older men and women had increased physical activity when having contact with not-close partners, but this link was stronger for men. This study advances our understanding of gender differences in older adults’ social experiences and well-being.

